# Book Review

**Published:** 1905-12

**Authors:** 


**Errors of Refraction and their Treatment.** By CHARLES BLAIR, M.D. Pp. 103. Bristol: John Wright & Co. 1905.—To those who want a clinical pocket-book whilst working at refraction we can cordially commend this little volume. It is concise, and it is lucid. No unnecessary details are introduced, and although it closely resembles the chapters on refraction in various text-books, many practical details and useful hints are furnished. A few pages at the end of the book are devoted to examples illustrating the text. The book is certainly convenient for its purpose both in size and arrangement.

**Laryngeal Phthisis.** By RICHARD LAKE, F.R.C.S., and HAROLD BARWELL. Second Edition. Pp. xi., 120. London: Baillière, Tindall & Cox. 1905.—Such a well-written and beautifully-illustrated resumé of the symptoms and treatment of laryngeal tuberculosis has naturally called for a second edition. There is little in the book that may not be found in the current text-books on diseases of the throat, except the full description of the surgical methods practised by the authors. The growing feeling on the part of most of the leading laryngologists that active surgical interference with laryngeal tuberculosis should be strictly limited to a relatively small class of cases, compels the belief that the special advocacy of operative measures is undesirable. With this exception, we have pleasure in recommending this monograph to all practitioners.

**Mucous Membranes: Normal and Abnormal.** By W. Stuart-Low, F.R.C.S. Pp. vii., 59. London: Baillière, Tindall, & Cox. 1905.—This little monograph, which was originally a lecture given before the students of the Polyclinic College, is mainly a description of the mucous membrane throughout the body, and is such as may be obtained direct from ordinary text-books of anatomy and physiology. But it is worthy of note that mucin has some power as a germicide, and the author refers to his own researches proving its bactericidal properties, and to the work of Arloing, which confirm his own views. We should feel more impressed with the author's views if he had not wandered off on the subject of hypomyxia as a cause of cancer. Why does malignant disease attack and spread in the mammary gland? How simple the explanation, for "in this gland there are no mucous cells or glands whatever!" But amidst all the padding, and, as it seems to us, nonsense, we believe that the author has done good service in directing attention to the value of mucin as a local application in such troublesome complaints as atrophic rhinitis and pharyngitis sicca.

**The Rational Treatment of Running Ears.** By F. Faulder White, F.R.C.S. Pp. 35. London: Iliffe & Sons. 1905.—As stated in the preface, the object of this little book is to show the excellent results which the author has obtained in the treatment of chronic suppurative otitis by an operation which he terms otectomy, followed by frequent and copious irrigations. The operation consists in removing the membrana tympani and malleus always, and the incus usually. For the subsequent irrigation a solution of various silico-fluorides is recommended. The writer apparently believes that other surgeons have been unsuccessful in curing otorrhœa by the operations of ossiculectomy and myringectomy, because these operations have been less thorough in scope than his own otectomies. He writes: "Anyone who takes the trouble to study my reported cases will see for himself that otectomy has been proved to be the most safe and most satisfactory of all operative measures hitherto undertaken for the cure of suppurative otitis." The technique of otectomy is imperfectly described, and no illustrations are given of certain instruments devised and used by the author, and considered to be essential. We think we may safely say that all otologists of experience have tried operations hardly distinguishable except in name from otectomy as here described, and that the results have hitherto been found for the most part less certain and satisfactory than those obtained from the radical mastoid operation. It is obvious that such otologists will not be justified in supporting Mr. Faulder White's contentions until they have proved for themselves by actual experience that results at least approaching in degree of success those reported by him are obtainable by his methods. That ossiculectomy or otectomy, call it what you will, is a less serious operation than the radical mastoid operation few will be found to deny, but we are of opinion that the author exaggerates the dangers and disadvantages of the latter proceeding in his desire to impress the reader with the advantages offered by the former. No doubt some of those into whose hands this booklet comes will be induced by the author's strong advocacy of the lesser operation to try it again, and subsequently to give more attention to thorough irrigation of the ear than they have done in the past. The object of both operations is the same, and the profession in the end will undoubtedly favour that mode of operating which achieves the object in view with the least disturbance of the affected organ and its surroundings.

**The Child's Diet.** By J. Sadler Curgenven. Pp. 96. London: H. K. Lewis. 1905.—This handy little volume contains a very sound and useful summary of the main principles of dietary in childhood. Infant feeding, concerning which the author has little new to say, is but briefly dealt with, the main portion of the book being devoted to the consideration of diet for older children. Dr. Curgenven is a strong supporter of the views recently expressed by Dr. Harry Campbell on the subject of mastication. He denounces the age of pap, and urges parents to give children food which they can bite, and to see that they do it. He also would reduce the amount of starchy food given to children of three or four years of age; this to be replaced by fat. In fact, he regards soft, easily-swallowed carbohydrates as the curse of modern dietetics, leading as they do to habits of imperfect mastication and insalivation. The two main difficulties in applying this principle are the higher cost of fatty foods and the curious repugnance to them which many children exhibit. The book contains chapters on the diet for children suffering from gastro-intestinal derangements, and also some very useful tables differentiating the starchy "foods" from those in which the starch is predigested or absent. There are also some useful recipes. For a book of this kind we think that rather too much space has perhaps been given to the description of pathological processes underlying digestive disturbances. These passages are, of course, intended for the lay reader; but even so should bile be described as the "natural antiseptic of the gastro-intestinal tract," or fats be said to "thicken the secretion from the liver"? These are, however, but minor points, and the practical advice given is on the whole excellent. The senior student even might derive many useful hints from its pages, as it deals very soundly with a subject which is apt to be something of a bugbear to him at the outset of his professional career.

